# Sestrin2 Signaling Pathway Regulates Podocyte Biology and Protects against Diabetic Nephropathy

**DOI:** 10.1155/2023/8776878

**Published:** 2023-02-10

**Authors:** Moein Ala

**Affiliations:** School of Medicine, Tehran University of Medical Sciences (TUMS), Tehran, Iran

## Abstract

Sestrin2 regulates cell homeostasis and is an upstream signaling molecule for several signaling pathways. Sestrin2 leads to AMP-activated protein kinase- (AMPK-) and GTPase-activating protein activity toward Rags (GATOR) 1-mediated inhibition of mammalian target of rapamycin complex 1 (mTORC1), thereby enhancing autophagy. Sestrin2 also improves mitochondrial biogenesis via AMPK/Sirt1/peroxisome proliferator-activated receptor-gamma coactivator *1 alpha* (PGC-1*α*) signaling pathway. Blockade of ribosomal protein synthesis and augmentation of autophagy by Sestrin2 can prevent misfolded protein accumulation and attenuate endoplasmic reticulum (ER) stress. In addition, Sestrin2 enhances P62-mediated autophagic degradation of Keap1 to release nuclear factor erythroid 2-related factor 2 (Nrf2). Nrf2 release by Sestrin2 vigorously potentiates antioxidant defense in diabetic nephropathy. Impaired autophagy and mitochondrial biogenesis, severe oxidative stress, and ER stress are all deeply involved in the development and progression of diabetic nephropathy. It has been shown that Sestrin2 expression is lower in the kidney of animals and patients with diabetic nephropathy. Sestrin2 knockdown aggravated diabetic nephropathy in animal models. In contrast, upregulation of Sestrin2 enhanced autophagy, mitophagy, and mitochondrial biogenesis and suppressed oxidative stress, ER stress, and apoptosis in diabetic nephropathy. Consistently, overexpression of Sestrin2 ameliorated podocyte injury, mesangial proliferation, proteinuria, and renal fibrosis in animal models of diabetic nephropathy. By suppressing *transforming growth factor beta* (TGF-*β*)/Smad and Yes-associated protein (YAP)/transcription enhancer factor 1 (TEF1) signaling pathways in experimental models, Sestrin2 hindered epithelial-mesenchymal transition and extracellular matrix accumulation in diabetic kidneys. Moreover, modulation of the downstream molecules of Sestrin2, for instance, augmentation of AMPK or Nrf2 signaling and inhibition of mTORC1, has been protective in diabetic nephropathy. Regarding the beneficial effects of Sestrin2 on diabetic nephropathy and its interaction with several signaling molecules, it is worth targeting Sestrin2 in diabetic nephropathy.

## 1. Background

Diabetic nephropathy is a major health concern with a growing incidence [1, 2]. Worldwide, its incidence has increased by 74% between 1990 and 2017 [1]. In addition, 2.62 million new cases and 134.58 million patients with diabetic nephropathy were identified in 2019 [2]. Globally, 405.99 thousand deaths and 13.09 million disability-adjusted life years (DALYs) have been attributed to chronic kidney disease (CKD) diabetes only in 2019 [2]. Patients with diabetic nephropathy are at a greater risk of cardiovascular adverse events, retinopathy, neuropathy, and other complications of diabetes [3].

Diabetic nephropathy is characterized by interstitial fibrosis, tubular atrophy, arteriolar hyalinosis, thickening of glomerular basement membrane (GBM), mesangiolysis, and mesangial expansion in pathology, even in the early phase of the disease [4]. Well-established diabetic nephropathy also leads to persistent proteinuria [3]. Furthermore, urinary albumin levels, nodular lesions, and mesangiolysis are associated with the rapid decline of the estimated glomerular filtration rate (eGFR) in patients with diabetic nephropathy [5]. The most important histological alterations in diabetic nephropathy are mesangial cell dysfunction and podocyte loss. Any attempt to improve diabetic nephropathy must be able to prevent these changes [6, 7], as previous studies mainly investigated the effects of different treatment modalities on these cells [6].

Currently, management of diabetic nephropathy is based on blood pressure control by angiotensin receptor blocker (ARB)/angiotensin-converting enzyme (ACE) inhibitors, lipid-lowering by statins, precise glycemic control, weight loss, salt restriction, and smoking cessation [3]. However, there is a lack of specific treatment for diabetic nephropathy, and numerous studies attempted to introduce a new strategy for treating diabetic nephropathy. Recently, it has been shown that upregulation of Sestrin2 can reverse the aforementioned pathological changes in diabetic nephropathy and maintain the viability and function of podocytes, mesangial cells, and tubular cells [8–10]. Here, it will be discussed how Sestrin2 is involved in the pathogenesis of diabetic nephropathy and whether targeted therapy for Sestrin2 can improve the outcome of the disease.

## 2. The Function of Sestrin2 in Cellular Physiology

Sestrin2 is a stress-inducible molecule and conserved antioxidant involved in the pathogenesis of several metabolic diseases [11]. Primarily, Sestrin2 was identified as a downstream of the renowned tumor suppressor, p53; however, it was then indicated that Sestrin2 can be controlled by p53-independent mechanisms [12, 13]. Sestrin2 is also a well-known inhibitor of the mammalian target of rapamycin complex 1 (mTORC1). mTORC1 activation promotes ribosomal protein synthesis and attenuates autophagy [11]. mTORC1 inhibition is one of the main mechanisms responsible for the cellular and metabolic effects of Sestrin2 [11]. Sestrin2 can activate AMP-activated protein kinase (AMPK)/tuberous sclerosis complex 2 (TSC2) pathway or inhibit GTPase-activating protein activity toward Rags (GATOR) 2/GATOR1/RagB pathway to control mTORC1 function [11, 14–16].

**Figure 1:**
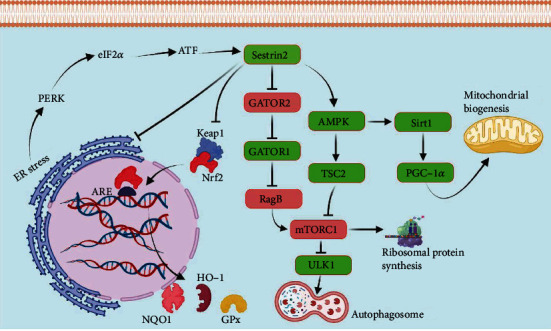
The involvement of Sestrin2 in cellular signaling. Sestrin2 is a central regulator of cellular homeostasis. Sestrin2 can liberate Nrf2 from Keap-1 sequestration and potentiate antioxidant defense. Sestrin2 can also inhibit mTORC1 through AMPK/TSC2 or GATOR2/GATOR1/RagB pathways. mTORC1 inhibition by Sestrin2 blocks ribosomal protein synthesis but activates ULK-1-mediated autophagy. Inhibition of ribosomal protein synthesis and activation of autophagy can prevent ER stress. However, ER stress can upregulate Sestrin2 through PERK/eIF2*α*/ATF pathway. Furthermore, activation of AMPK/Sirt1/PGC-1*α* pathway by Sestrin2 is deeply involved in mitochondrial biogenesis.

AMPK/TSC2 pathway closely interacts with mTORC1 to maintain cell viability during energy deprivation [17]. By phosphorylating AMPK, Sestrin2 indirectly activates TSC2, a tumor suppressor, thereby inhibiting mTORC1 [15, 18]. Similarly, GATOR2/GATOR1/RagB regulates mTORC1 based on amino acid availability [19]. In the presence of sufficient amino acids, GATOR2/GATOR1/RagB pathway activates mTORC1-mediated ribosomal translation and cell growth [19]. Sestrin2 can inhibit GATOR2, thereby liberating GATOR1. GATOR1 is a GTPase-activating protein for RagB, keeping it in its inactive form [16]. GATOR1 release inhibits RagB and eventually attenuates mTORC1 signaling pathway [16]. Sestrin2 is also a leucine sensor for mTORC1, i.e., in the presence of a sufficient amount of leucine, this amino acid binds to Sestrin2, abolishing its inhibitory effect on GATOR2 and activating mTORC1 (Figure 1) [20].

mTORC1 is a major inhibitor of autophagy [21]. Inhibition of mTORC1 can markedly potentiate autophagy by activating autophagy-related 13 (ATG13)/Unc-51-like autophagy activating kinase (ULK1) signaling pathway [22]. In addition to its effects on autophagy, Sestrin2 can reduce endoplasmic reticulum (ER) stress, a major driver of inflammation and cell death [8]. Harmful stimuli and uncontrolled oxidative stress can damage DNA and protein structure and stimulate ER stress, which can lead to apoptosis in severe cases [23]. Sestrin2 overexpression markedly suppresses ER stress, whereas Sestrin2 knockdown significantly aggravates ER stress [8, 24, 25]. Sestrin2-mediated AMPK/mTORC1 pathway activation can effectively suppress ER stress [24]. In the absence of Sestrin2, overactivation of mTORC1 leads to expedited ribosomal translation, which facilitates the accumulation of misfolded polypeptides in inflammation [25]. Eventually, misfolded polypeptide accumulation ignites ER stress and damages cell structure, known as the misfolded protein response [25, 26]. Interestingly, ER stress can upregulate Sestrin2 through protein kinase RNA-like endoplasmic reticulum kinase (PERK)/eukaryotic initiation factor-2 alpha (eIF2*α*)/activating transcription factor 4- (ATF4-) mediated signaling pathway [25, 27, 28]. Kim et al. elucidated that deletion or insufficient expression of PERK, EIF2*α*, and ATF4 impairs the compensatory upregulation of Sestrin2 during ER stress [27]. Oxidative stress also potentiates ER stress and directly or indirectly endangers cellular viability [28]. ER stress promotes the expression of Sestrin2, thereby accelerating nuclear factor erythroid 2-related factor 2 (Nrf2) release from Kelch-like ECH-associated protein 1 (Keap1) sequestration and strengthening antioxidant defense [28]. Nrf2 is a major transcription factor whose activation can enhance the expression of numerous endogenous antioxidants; therefore, ER stress may ameliorate uncontrolled oxidative stress by upregulating Sestrin2/Nrf2 pathway [23, 29]. Therefore, ER stress can promote Sestrin2 expression as a compensatory mechanism to attenuate both oxidative stress and ER stress (Figure 1) [27].

Sestrin2 facilitates sequestosome 1 (SQSTM1) mitochondrial preparation for recognition by autophagic machinery [30]. Furthermore, Sestrin2 enhances the autophagic degradation of mitochondria by activating ULK1 [30, 31]. Consistently, Sestrin2 deletion leads to insufficient mitophagy and mitochondrial dysfunction, oxidative stress, and cell death in stress conditions [32].

As an activator of AMPK, Sestrin2 is deeply involved in mitochondrial biogenesis. Sestrin2 activates AMPK/peroxisome proliferator-activated receptor gamma coactivator 1-alpha (PGC-1*α*) pathway, which improves mitochondrial biogenesis [33]. Sestrin2 silencing with a siRNA impaired mitochondrial biogenesis in rats [33]. Likewise, Lin et al. revealed that downregulation of Sestrin2 leads to mitochondrial dysfunction in cultured podocytes and rat kidney. In comparison, upregulation of Sestrin2 enhanced AMPK signaling pathway and improved mitochondrial biogenesis [9].

Sestrin2-mediated protection against oxidative stress mainly relies on Nrf2/antioxidant responsive element (ARE) signaling pathway [34]. Sestrin2 expedites ULK1-dependent phosphorylation of P62, which accelerates autophagic degradation of P62 and its target, Keap-1 [35, 36]. Autophagic degradation of Keap1 potentiates Nrf2 function and increases the expression of Sestrin2 and other antioxidants [37]. Sestrin2 depletion remarkably weakens antioxidant defense, while Sestrin2 overexpression strengthens Nrf2/ARE signaling pathway for reactive oxygen species (ROS) scavenging [34]. Thus, there is a mutual and positive control mechanism between Sestrin2 and Nrf2 (Figure 1).

### 2.1. The Function of Nrf2 and Its Association with Sestrin2

Nrf2 is a transcription factor and a master antioxidant molecule that protects cell viability in response to stress signals [38]. Upon activation and nuclear translocation, Nrf2 binds to the antioxidant response element (ARE) and promotes the expression of numerous antioxidants such as Sestrin2, heme oxygenase 1 (HO-1), NAD(P)H:quinine oxidoreductase 1 (NQO1), superoxide dismutase (SOD), thioredoxin reductase 1 (Txnrd1), and glutathione peroxidase (Gpx) [39–43]. Normally, Nrf2 is sequestered by Keap1, leading to its ubiquitination and degradation, undermining its antioxidant function [38, 44]. In response to stress signals, p62-dependent autophagic degradation of Keap1 releases the sequestered Nrf2, which will be translocated into the nucleus afterward [38]. Deletion of Nrf2 or pharmacological inhibition of Nrf2/ARE pathway weakens the antioxidant defense and aggravates oxidative stress and inflammation [43, 45]. Conversely, Nrf2 upregulation or Keap1 deletion potentiates antioxidant defense and attenuates inflammation [46, 47]. In addition, Nrf2 can bind to ARE in the promoter region of nonantioxidant genes. For instance, Nrf2 can bind to the ARE in the promoter region of autophagy-related genes such as nuclear dot protein 52 (NDP52) and Ca^2+^/calmodulin-dependent protein kinase II *α* (CaMKII*α*), thereby promoting autophagy [48–50]. Consistently, it has been shown that deletion of Nrf2 suppresses autophagy [49]. The primary function of Nrf2 is ascribed to its antioxidant property; however, it seems that Nrf2 is also involved in other dimensions of cellular homeostasis, such as autophagy [51].

### 2.2. Sestrin2 Ameliorates Oxidative Stress in Diabetic Nephropathy

Hyperglycemia ignites oxidative stress [52]. Even an acute episode of hyperglycemia can increase the plasma levels of nitrotyrosine, a marker of oxidative stress, in healthy subjects [53]. Several mechanisms such as mitochondrial dysfunction, accumulation of advanced glycation end products (AGE), and activation of protein kinase C have been implicated in diabetes-associated oxidative stress [54, 55]. Unleashed oxidative stress damages cellular macromolecules, expedites mitochondrial dysfunction, and leads to lipid peroxidation, DNA breakage, and accumulation of advanced oxidation protein products (AOPP) [54]. These alterations also provoke an inflammatory response and cause more severe oxidative stress [54]. Khazim et al. indicated that podocytes exposure to high glucose concentration results in 60% increase in intracellular superoxide concentration, 90% increase in NADPH oxidase activity, 100% increase in NADPH oxidase 4 (NOX4) expression, and 150% increase in apoptotic podocyte death [56]. High glucose levels also downregulate antioxidants such as GSH and SOD and weaken antioxidant defense in podocytes [57].

**Figure 2:**
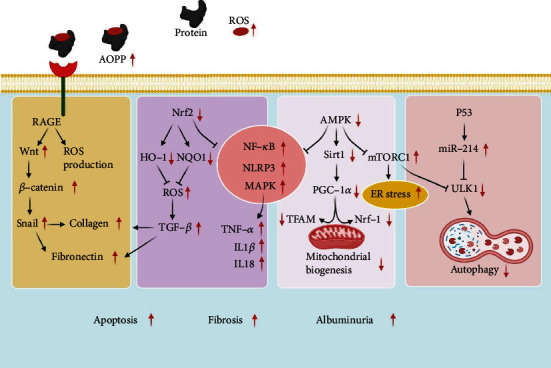
Impaired signaling pathway in diabetic nephropathy. Overproduction of ROS and subsequent release of AOPP activate RAGE, which exacerbates ROS production and accelerates renal fibrosis by upregulating Wnt/*β*-catenin/Snail pathway and TGF-*β*/Smad pathway. Patients with diabetic nephropathy simultaneously suffer from an insufficient antioxidant defense due to impaired Nrf/HO-1/NQO1 pathway. Downregulation of AMPK/Sirt1/PGC-1*α* in diabetic nephropathy undermines mitochondrial biogenesis. Furthermore, the inhibitory effect of Nrf2 and AMPK on inflammatory signaling molecules such as NF-*κ*B, NLRP3, and MAPK is weakened in diabetic nephropathy. Moreover, overactivation of mTORC1 and P53/miR-214 inhibits ULK-1 and autophagy in diabetic nephropathy. The outcome of these molecular alterations is apoptosis, albuminuria, and fibrosis.

Blockade of AOPP signaling by inhibiting receptor of advanced glycation end products (*α*RAGE) remarkably reversed the harmful effect of diabetic nephropathy serum on podocytes [58]. It was found that both serum-derived and exogenous AOPP upregulated Wnt1 and Wnt7a expressions, induced *β*-catenin signaling pathway, reduced podocyte markers, and increased fibrogenesis in mice or cell culture [59]. Wnt blockade or *β*-catenin knockdown in podocytes downregulated profibrotic factors such as Snail, matrix metalloproteinase 9, fibronectin, and desmin (Figure 2) [59]. Furthermore, podocyte-specific ablation of *β*-catenin protected against AOPP-mediated podocyte injury and albuminuria in mice [59]. Likewise, it was shown that AOPP upregulate FOXO3a by inhibiting its autophagic degradation through ROS/mTORC1 signaling pathway [58]. FOXO3a finally stimulates podocyte apoptotic death [58].

Interestingly, NOX4 deletion or inhibition in a mouse model of diabetic nephropathy or NOX4 silencing in human podocytes markedly suppressed oxidative stress, inflammation, profibrotic factor expression, and enhanced podocyte marker expression. In addition, attenuation of NOX4 function improved albuminuria and structural alterations such as glomerular sclerosis, basement membrane thickening, and mesangial area expansion in diabetic mice [60, 61].

Sestrin2 overexpression ameliorated oxidative stress in the kidney of diabetic mice associated with both functional and structural improvement [62]. Similarly, Sestrin2 overexpression attenuated oxidative stress in human mesangial cell exposed to high glucose [62]. Sestrin2 overexpression also attenuated oxidative stress and apoptosis in murine podocyte culture exposed to high glucose [63]. Using a mouse model of diabetic nephropathy, it was shown that attenuation of oxidative stress by Sestrin2 overexpression reversed high glucose-mediated podocyte loss and podocyte malfunctioning [63]. High glucose exposure downregulated podocyte markers such as synaptopodin and nephrin in the kidney of diabetic mice, while Sestrin2 overexpression maintained their expression [63].

These findings suggest that attenuation of overwhelming oxidative stress by Sestrin2 may halt the structural and functional alteration in diabetic nephropathy.

### 2.3. Sestrin2 Improves Autophagy in Diabetic Nephropathy

Autophagy is a cellular mechanism that can remove dysfunctional organelles and misfolded proteins and maintain normal cellular homeostasis [64, 65]. It has been found that patients and animals with diabetic nephropathy have decreased or impaired autophagy in their kidneys [66–68]. Impaired autophagy, accumulation of dysfunctional lysosomes, and apoptosis were found in cultured podocytes exposed to serum from diabetic patients and in diabetic rats with massive proteinuria [66].

Autophagy also hinders epithelial-mesenchymal transition in podocytes [69]. Augmentation of autophagy prevented podocyte loss, tubular cell apoptosis, and renal fibrosis in diabetic mice [70]. In particular, mTORC1 inhibition restores autophagy and protects against high glucose-induced mesangial cell dysfunction. In contrast, inhibition of autophagy by 3-methyladenine (3-MA) exacerbates mesangial and glomerular cell dysfunction and induces apoptosis and intercellular matrix protein deposition [71].

Autophagy-related gene 7 (Atg7) knockout in the proximal convoluted tubules led to tubular damage, inflammation, albuminuria, renal hypertrophy, and fibrosis in diabetic mice [67]. Furthermore, it was observed that high glucose concentrations downregulate ULK1 in diabetic mice [67]. In fact, P53 upregulates miR-214 to decrease ULK1 expression in diabetic nephropathy [67]. It was indicated that patients with diabetic nephropathy have a significantly lower expression of ULK1 and a higher expression of P53 and miR-214 [67]. Moreover, the expression levels of P53 and miR-214 negatively correlated with the expression levels of ULK1 in those patients (Figure 2) [67]. Interestingly, Wang et al. revealed that ineffective autophagic flux prevents P62-dependent degradation of zonula occludens-1 (ZO-1), a tight junction protein, and accelerates slit diaphragm replacement by tight junction in podocytes, causing foot process effacement [72].

Previously, it has been observed that overexpression of Sestrin2 in hypoxic renal tubular cells can enhance autophagosome formation and autophagy and protect against apoptosis [12]. Similarly, activation of Sestrin2 has been associated with autophagosome formation and autophagy in immortalized human embryonic kidney cells, effectively accelerated degradation of dysfunctional mitochondria, and ameliorated Cu-induced oxidative stress [31].

Given the impaired autophagic flux in patients with diabetic nephropathy, the protective effect of autophagy on podocyte loss and kidney injury, and autophagy activation by Sestrin2, it can be assumed that Sestrin2 ameliorates diabetic nephropathy.

### 2.4. Sestrin2 Improves Mitochondrial Biogenesis and Mitophagy in Diabetic Nephropathy

High glucose-related mitochondrial dysfunction is strongly linked to podocyte injury in diabetic nephropathy [73]. Insufficient mitochondrial biogenesis can immensely decrease podocyte viability and contribute to proteinuria [74]. Renal interstitial fibrosis has been positively correlated with urinary supernatant mitochondrial DNA levels and negatively correlated with intrarenal mitochondrial DNA levels in 92 patients with biopsy-proven diabetic nephropathy [75]. Furthermore, urinary supernatant mitochondrial DNA levels have been inversely correlated with eGFR in these patients [75]. Diabetic nephropathy causes insufficient mitophagy to remove dysfunctional mitochondria, which aggravates albuminuria, renal tubular injury, and interstitial fibrosis [76]. Conversely, potentiation of mitophagy can markedly improve renal function and inhibits epithelial-mesenchymal transition in the kidney [76].

Augmentation of mitochondrial biogenesis and mitophagy through AMPK/Sirt1/peroxisome proliferator-activated receptor-gamma coactivator *1 alpha* (PGC-1*α*) signaling pathway has been protective against proteinuria and podocyte injury in a mouse model of diabetic nephropathy [73]. Interestingly, it was shown that mitochondrial transfer from mesenchymal stem cells to macrophages can activate PGC-1*α*, attenuate inflammation, and alleviate diabetic nephropathy in mice [77]. Consistently, inhibition of AMPK or Sirt1 or downregulation of PGC-1*α* with a siRNA significantly reduced ATP production and downregulated the expression of mitochondrial biogenesis markers such as Nrf-1 and mitochondrial factor A (TFAM) in a mouse model of diabetic nephropathy. TFAM protects mitochondrial DNA integrity in diabetic nephropathy (Figure 2) [78]. Moreover, it has been unveiled that patients with diabetic nephropathy have significantly lower expression levels of PGC-1*α* in their kidney biopsies than nondiabetic individuals [79]. Similarly, lower expression levels of Sirt1 predicted albuminuria in patients with diabetic nephropathy [80].

Diabetic nephropathy is also associated with increased mitochondrial fission [81, 82]. Patients with diabetic nephropathy have more fragmented mitochondria in their podocytes [82]. Furthermore, the mitochondrial aspect ratio value is inversely correlated with urine protein levels in these patients [82]. High glucose concentrations downregulates dual-specificity protein phosphatase-1 (DUSP1) [83]. Lower levels of DUSP1 activate c-Jun N-terminal kinases (JNK), which selectively phosphorylates mitochondrial fission factor (Mff), exacerbates oxidative stress, and accelerates apoptotic cell death in diabetic nephropathy [83]. Attenuation of mitochondrial fission with augmentation of mitophagy can improve renal dysfunction, glomerular apoptosis, and renal fibrosis in diabetic mice [84].

Lin et al. indicated that mitochondrial dysfunction and podocyte injury in diabetic rats are associated with marked downregulation of Sestrin2 [9]. Consistently, plasmid-mediated upregulation of Sestrin2 improved mitochondrial dysfunction in podocytes exposed to high glucose concentrations through AMPK activation [9]. In contrast, plasmid-mediated downregulation of Sestrin2 further suppressed AMPK function and exacerbated mitochondrial dysfunction and apoptosis in podocytes exposed to high glucose concentrations [9]. Furthermore, overexpression of Sestrin2 was shown to enhance mitophagy and accelerated removal dysfunctional mitochondria in hypoxia- and Cu-mediated renal tubular cell damage [12, 31]. It has been shown that Sestrin2 colocalizes and interacts with *adenosine triphosphate 5A* (ATP5A) in the mitochondrion to induce mitophagy in HEK293 cells, and Sestrin2 inhibition attenuated mitophagy in these cells [31].

Regarding the importance of mitochondrial dysfunction in the pathogenesis of diabetic nephropathy and the involvement of Sestrin2 in mitochondrial homeostasis, targeting Sestrin2 expression may improve diabetic nephropathy.

### 2.5. Sestrin2 Can Hinder ER Stress in Diabetic Nephropathy

Accumulation of misfolded proteins leads to unfolded protein response to maintain ER homeostasis [85]. Severe ER stress can endanger cellular viability and activate cell death mechanisms [85]. Severe ER stress also induces autophagy which may be the last resort to remove the dysfunctional ER and restore cell damage [86]. Consistently, it has been shown that modulation and prevention of severe ER stress by a chemical chaperone can improve diabetic nephropathy and renal fibrosis in diabetic rats [87]. Patients with diabetic nephropathy have more severe ER stress in their kidneys [88]. It was shown that albumin and high glucose both induce ER stress in human renal tubular cells in vitro [89]. In addition, stimulation of RAGE by AGEs was shown to robustly induce ER stress in proximal tubular cells, attenuated by blocking RAGE signaling pathway [90]. Similarly, Chiang et al. observed that AGEs ignite ER stress in murine glomerular mesangial cell line [91]. They found that inhibition of ER stress mitigates mesangial cells apoptosis and inhibition of autophagy exacerbates mesangial cell loss [91].

mTORC1 activation is heavily involved in ER stress [92]. Previously, it was unveiled that inhibiting mTORC1 by rapamycin ameliorates high glucose-induced ER stress, subsequently preventing podocyte injury [92]. mTORC1 activation can deteriorate ER stress by two major mechanisms: first, by amplification of ribosomal protein synthesis and overproduction of misfolded proteins during cell stress and, second, by inhibition of autophagic removal of misfolded proteins and dysfunctional ER (Figure 2) [93–95]. Jia et al. indicated that Sestrin2 downregulation expedites albumin-stimulated HK-2 cell epithelial-mesenchymal transition and ER stress, whereas Sestrin2 upregulation hinders epithelial-mesenchymal transition and ER stress in HK-2 cell [8].

Therefore, Sestrin2-mediated inhibition of mTORC1 and ER stress may improve renal fibrosis in diabetic nephropathy.

## 3. The Beneficial Effects of Sestrin2 on the Kidney and Diabetic Nephropathy

Sestrin2 can protect against several metabolic diseases and is the master of a compensatory mechanism to preserve the damaged organ [11]. Previously, it has been shown that Sestrin2 alleviates renal oxidative stress, thereby maintaining normal blood pressure [96]. Consistently, it was revealed that silencing Sestrin2 in the renal proximal tubular cells enhances the production of hyperoxidized peroxiredoxins and ROS and expedites lipid peroxidation [96]. Similarly, Sestrin2 silencing with small interfering RNA increased renal oxidative stress and blood pressure in mice [96]. Furthermore, Sestrin2 overexpression in renal tubular cells strongly enhanced autophagy and protected against acute kidney injury induced by renal ischemia/reperfusion in rats [12].

The expression of Sestrin2 markedly decreased in adriamycin-induced nephropathy [97]. Reduced expression of Sestrin2 in adriamycin-induced nephropathy was followed by upregulation of mTORC1/phosphorylated S6 ribosomal protein- (P-S6RP-) mediated ribosomal protein synthesis, glomerulosclerosis, severe periglomerular fibrosis, and proteinuria in rats [97]. Further decrease in Sestrin2 expression by short hairpin RNA vigorously potentiated mTORC1/P-S6RP axis and led to apoptosis in cultured parietal epithelial cells [97].

Compared with healthy subjects, patients with diabetic nephropathy had significantly lower serum levels of Sestrin2, and the difference in the serum levels of Sestrin2 increased with albuminuria [10]. Additionally, there was a negative correlation between the serum levels of neutrophil gelatinase-associated lipocalin (NGAL), an indicator of diabetic nephropathy, and the serum levels of Sestrin2 in diabetic patients [10]. The inverse correlation between the serum levels of NGAL and Sestrin2 was more robust in those with macroalbuminuria, followed by those with microalbuminuria and without albuminuria [10].

Previously, it was found that patients with diabetic nephropathy have markedly decreased expression of Sestrin2 in their kidneys compared with their nondiabetic subjects [9, 63]. In particular, patients with diabetic nephropathy have reduced expression of Sestrin2 in their podocytes [9]. It was revealed that Sestrin2 expression also decreases in the kidney of rats in streptozotocin-induced diabetes model [9]. Furthermore, high glucose exposure downregulated Sestrin2 expression in cultured podocytes [9], and decreased expression of Sestrin2 exacerbated mitochondrial dysfunction in podocytes exposed to high glucose [9]. By upregulating AMPK, Sestrin2 modulated mitochondrial dysfunction, oxidative stress, and apoptosis in podocytes exposed to high glucose [9].

**Figure 3:**
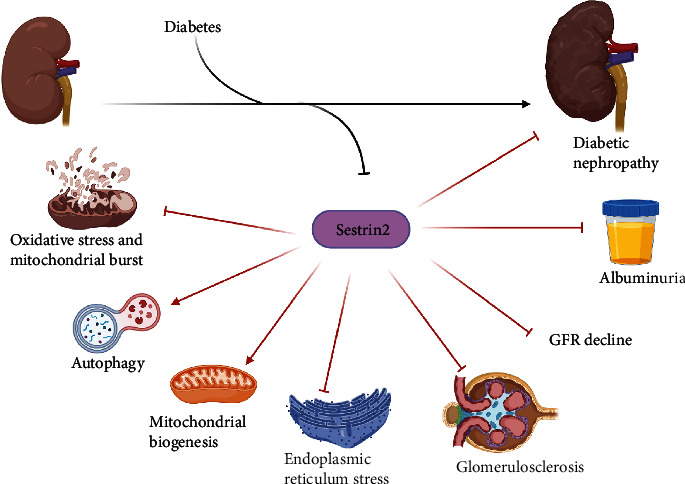
The involvement of Sestrin2 in diabetic nephropathy. Diabetes and hyperglycemia downregulate Sestrin2 expression in the kidney. Patients and animals with diabetic nephropathy have decreased expression of Sestrin2 in their kidneys which is associated with progression of albuminuria and GFR decline. Sestrin2, an antioxidant and a metabolic regular, enhances autophagy and mitochondrial biogenesis and attenuates oxidative stress and endoplasmic reticulum stress. Furthermore, Sestrin2 prevents fibrogenesis. It has been observed that upregulation of Sestrin2 can suppress inflammation, oxidative stress, and renal fibrosis in animal models of diabetic nephropathy and decelerate the progression of albuminuria and GFR decline.

Compared with Sesn2^+/+^, Sesn2^−/−^ led to more severe features of glomerular fibrosis, including mesangial matrix hypertrophy and fibronectin and collagen IV deposition in high-fat diet-fed mice [98]. Besides, Sesn2^−/−^ was associated with podocyte loss and decreased expression of synaptopodin, a marker of differentiated podocytes [98]. Similarly, Bian et al. and Song et al. indicated that Sestrin2 overexpression could ameliorate renal function and decrease creatinine, blood urea nitrogen, and albuminuria in diabetic mice [62, 63]. Overexpression of Sestrin2 also attenuated oxidative stress, reduced *transforming growth factor beta* (TGF-*β*) expression, and prevented glomerular hypertrophy, mesangial expansion, and extracellular matrix deposition in diabetic mice in these studies (Figure 3) [62, 63].

High glucose concentration upregulates *NOX4* and increases ROS and peroxynitrite generation in the kidney [99]. It was found that peroxynitrite accumulation leads to endothelial nitric oxide synthase (eNOS) uncoupling and dysfunction. eNOS uncoupling aggravates oxidative stress, decreases nitric oxide (NO) production, and increases fibronectin deposition in the kidney [99]. Interestingly, Sestrin2/AMPK pathway activation inhibits NOX4-mediated eNOS uncoupling, attenuates oxidative stress, and prevents fibronectin deposition in the kidney [99]. On the contrary, hyperglycemia downregulates Sestrin2/AMPK pathway in the glomerular mesangial cells, leads to eNOS uncoupling, exacerbates oxidative stress, and increases fibronectin deposition in the kidney [99].

Previously, it was found that patients with diabetic nephropathy have elevated levels of miR-4756 in their urinary extracellular vesicles [8]. Further investigation uncovered that miR-4756 downregulates the expression of Sestrin2 in the renal tubular epithelial cells and facilitates albumin-mediated epithelial-to-mesenchymal transition and ER stress [8]. In addition, Sestrin2 overexpression reversed the effects of miR-4756 on epithelial-to-mesenchymal transition and ER stress in cultured renal tubular epithelial cells [8].

Sestrin2 upregulation was shown to accelerate autophagic Keap1 degradation and improve Nrf2-mediated antioxidant response in a rat model of diabetic nephropathy and murine podocyte cell line exposed to high glucose concentration [100]. Sestrin2 also improved mitophagy and inhibited NLR family pyrin domain containing 3- (NLRP3-) mediated inflammatory response in cultured podocytes [100]. Hyperglycemia and hyperlipidemia can stimulate NLRP3 overactivity, which results in the production of several inflammatory cytokines such as IL1*β* and IL18 [101]. NLRP3 also strengthens other inflammatory pathways, such as NF-*κ*B signaling pathway and mitogen-activated protein kinase (MAPK) signaling pathway, and augments oxidative stress and inflammation in diabetic nephropathy [102].

As mentioned previously, Sestrin2 is a leucine-sensing molecule for mTORC1, and leucine bioavailability attenuates the inhibitory effect of Sestrin2 on mTORC1 [20]. Interestingly, it has been elucidated that increased expression of leucine-rich *α*-2 glycoprotein-1 (LRG1) worsens the outcome of diabetic nephropathy [103]. It was uncovered that the plasma levels of LRG1 are independently and positively associated with the progression of CKD and albuminuria in diabetic patients [104]. LRG1 is remarkably expressed in the glomerular endothelial cells and tubulointerstitial segment of nephrons in diabetic nephropathy [103, 105]. LRG1 vigorously activates TGF-*β*/Smad3 signaling pathway in the kidney and aggravates tubulointerstitial fibrosis [103, 105]. Global loss of LRG1 could significantly attenuate TGF-*β*/Smad3 signaling pathway and ameliorate tubulointerstitial fibrosis in mice [103]. Consistently, Song et al. indicated that Sestrin2 suppresses the thrombospondin 1 (TP-1)/TGF-*β*/Smad3 signaling pathway to prevent profibrotic changes in podocytes exposed to high glucose [63]. Compared with normal and decreased expression of Sestrin2 in podocyte culture, increased expression of Sestrin2 has been associated with lower expression of *α*-SMA and desmin and higher expression of E-cadherin, nephrin, and synaptopodin in podocytes after exposure to high glucose [63]. Sestrin2 overexpression also reduced the expression of bax, cleaved caspase-3, and NOX4 and increased the expression of bcl2 in podocytes exposed to high glucose [63]. Furthermore, Sestrin2 overexpression also reversed GBM thickening, extracellular matrix deposition, and foot process effacement in the mouse model of diabetic nephropathy (Figure 3) [63].

In addition to its beneficial effects on podocytes, overexpression of Sestrin2 hindered high glucose-induced mesangial proliferation, production of fibronectin, collagen IV, and ROS in human mesangial cell culture [62]. It was found that Sestrin2 can target Hippo signaling pathway in mesangial cells, thereby alleviating renal fibrosis in diabetic nephropathy [62]. In particular, Sestrin2 overexpression suppressed Yes-associated protein (YAP) and transcription enhancer factor 1 (TEF1) function, whose transcriptional functions are crucial for extracellular matrix protein expression [62]. Consistently, previous studies indicated that YAP and TEF1 are highly expressed in the kidney of diabetic patients. In addition, YAP positively correlates with creatinine, blood urea nitrogen, GFR decline, stage of diabetic nephropathy, progression of diabetic nephropathy in pathology, and systolic blood pressure [106, 107]. Furthermore, deletion or inhibition of YAP markedly hampered tubulointerstitial and glomerular fibrosis in diabetic mice [107].

Overall, Sestrin2 can remarkably reverse a group of abnormal signaling pathways observed in diabetic nephropathy and reverse the functional and structural alterations.

## 4. What is the Effect of Downstream Molecules of Sestrin2 on Diabetic Nephropathy?

### 4.1. The Beneficial Effects of Nrf2 on Diabetic Nephropathy

As mentioned previously, Nrf2 activation is the mainstay of antioxidant response [108]. It was found that compared with Nrf2^+/+^ mice, Nrf2^−/−^ mice experience more severe oxidative stress, apoptosis, and fibrotic change in acute kidney injury [109]. Similarly, it has been reported that compared with Nrf2^+/+^ mice, Nrf2^−/−^ mice experience more severe oxidative stress, kidney injury, and TGF-*β*-mediated renal fibrosis in a mouse model of streptozotocin-induced diabetic nephropathy [108]. Nrf2 overexpression dose dependently inhibited the promoter of TGF-*β*, whereas Nrf2 knockdown enhanced TGF-*β* signaling pathway in human mesangial cells and enhanced fibronectin production [108]. Nrf2/HO-1 axis activation and subsequent attenuation of oxidative stress strongly suppressed inflammation and downregulated intercellular adhesion molecule 1 (ICAM-1), vascular cell adhesion molecule 1 (VCAM-1), and inflammatory nitric oxide synthase (iNOS) in a mouse model of diabetic nephropathy [110]. Furthermore, Nrf2/HO-1 axis activation mitigated renal mesangial fibrosis and albuminuria in a mouse model of streptozotocin-induced diabetic nephropathy [110, 111]. Nrf2 activation can deeply attenuate the inflammatory signals relayed by major signaling molecules such as nuclear factor kappa B (NF-*κ*B) and NLRP3 [112–116]. The inhibitory effect of Nrf2 on NF-*κ*B and NLRP3 prevents the release of numerous inflammatory cytokines such as IL1*β*, interleukin 6 (IL6), and tumor necrosis factor *α* (TNF-*α*), thereby hindering apoptosis and kidney injury (Figure 2) [112–114, 116]. Downregulation of Keap1 by miR-200a also effectively enhanced Nrf2 function and protected against albuminuria, oxidative stress, and fibrosis in streptozotocin-induced diabetic nephropathy [111, 117, 118]. Conversely, inhibition of miR-200a-induced downregulation of Keap1 by LNA-200a abolished the protective effects of Nrf2 on diabetic nephropathy [111]. Regarding the effect of Sestrin2 on autophagic degradation of Keap1, enhancing Sestrin2 expression can simultaneously strengthen Nrf-2-mediated renal protection in diabetic nephropathy [100]. Nrf2 activation both in a Sestrin2-dependent or Sestrin2-independent manner can improve the outcome of diabetic nephropathy and decelerate disease progression.

### 4.2. The Effect of AMPK on Diabetic Nephropathy

AMPK is involved in cellular energy homeostasis and is activated by energy stress [119, 120]. Similar to Sestrin2, AMPK has been implicated in a wide variety of cellular mechanisms such as autophagy, mitochondrial biogenesis, redox regulation, inflammation, proliferation, and aging [119, 120]. As mentioned previously, Sestrin2 augments AMPK phosphorylation [14]. Insufficient AMPK phosphorylation results in metabolic perturbation of the kidney, eGFR decline, and renal fibrosis [121]. Hyperglycemia leads to decreased phosphorylation of AMPK and attenuates its function [122]. Direct activation of AMPK by potent small molecule activators of AMPK such as PF-06409577 and PF-249 significantly reduced albuminuria in a rat model of diabetic nephropathy [123]. AMPK activators markedly decreased urinary markers of kidney injury such as kidney injury molecule-1 (KIM-1), tissue inhibitor matrix metalloproteinase 1, urine albumin-creatinine ratio, *urine protein/creatinine ratio*, and thiobarbituric acid-reactive substances in the study [123]. In addition, AMPK activators increased PGC-1*α* expression and lowered collagen 1a1 deposition [123]. AMPK-mediated inhibition of mTORC1 significantly enhanced autophagy and greatly protected against podocyte injury in diabetic rats [124]. AMPK phosphorylation was also followed by upregulation of ULK1, enhancement of autophagy, and decrease in oxidative stress, podocyte loss, fibrosis, and albuminuria in a mouse model of diabetic nephropathy [125]. Conversely, inhibition of AMPK by compound C abolished the beneficial effects of AMPK in podocyte viability and aggravated high glucose-mediated oxidative stress, inflammation, apoptosis, collagen accumulation, and GMB thickening (Figure 2) [126, 127].

Decreased function of PGC-1*α* was shown to impair mitochondrial function and facilitate fibrogenesis in diabetic nephropathy [128]. Activation of AMPK/PGC-1*α* signaling pathway also attenuates oxidative stress and apoptosis in a mouse model of diabetic nephropathy and reduces albuminuria [129, 130]. Similarly, it was found that patients with diabetic nephropathy have lower expression levels of PGC-1*α* [88].

AMPK activation can profoundly suppress NADPH oxidase 4- (NOX-4-) mediated fibroblast activation in diabetic nephropathy [122]. He et al. unveiled that AMPK inhibition by hyperglycemia amplifies NOX-4-mediated ROS release and activates extracellular signal-regulated kinase (ERK) signaling pathway, which finally provokes fibroblast activation and proliferation in the kidney [122].

Furthermore, AMPK plays an important role in alleviating inflammation in diabetic nephropathy [131]. For instance, it was reported that AMPK activation can suppress NLRP3 overactivity and decrease inflammatory cytokines such as IL1*β*, IL6, IL18, and TNF-*α* in a mouse model of diabetic nephropathy [131, 132]. Similarly, AMPK inhibits NF-*κ*B signaling pathway in diabetic nephropathy, which is a major driver of the inflammatory response [132]. By binding to the receptor activator of NF-*κ*B, P65 promotes the expression of NOX-4 in podocytes exposed to high glucose concentrations (Figure 2) [133].

An increasing number of studies are showing that AMPK activation can protect against structural and functional damage in diabetic nephropathy. Besides the other benefits, higher expression of Sestrin2 can strengthen the protective effects of AMPK signaling pathway on diabetic nephropathy [99].

### 4.3. The Effect of mTORC1 on Diabetic Nephropathy

A substantial proportion of functions mediated by Sestrin2 are ascribed to mTORC1 inhibition [134]. Therefore, a deep insight into the involvement of mTORC1 in diabetic nephropathy is crucial to understanding the effect of Sestrin2 on the disease.

mTORC1 is heavily involved in the regulation of glomerular and podocyte homeostasis. Diabetic nephropathy is characterized by mTORC1 overactivity in the kidney biopsies from diabetic patients and diabetic mice [135, 136]. Interestingly, the rs7212142 polymorphism in mTORC1 gene has been significantly associated with diabetic nephropathy in the Chinese population [137]. The basal and physiologic function of mTORC1 is critical for the normal function of the kidney as it was shown that podocyte-specific deletion of mTORC1 leads to decreased adaptation to stress conditions and more severe glomerulosclerosis in diabetic mice [135]. On the other hand, mTORC1 overactivity accelerates glomerulosclerosis in diabetic nephropathy [135]. Podocyte-specific genetic inhibition of mTORC1 hyperactivation by heterozygous knockout of Raptor prevented mesangial matrix accumulation and glomerulosclerosis in diabetic mice (Figure 2) [135].

Overactivation of phosphoinositide 3-kinases (PI3K)/protein kinase B (AKT)/mTORC1 pathway by high concentrations of glucose is a major driver of renal fibrosis in diabetic nephropathy [138, 139]. mTORC1 overactivity promotes ROS production and activates apoptosis in the kidney of diabetic rats and glomerular mesangial cells culture exposed to high glucose [140]. mTORC1 inhibition by rapamycin significantly reverted these alterations [140]. It was unveiled that despite high glucose concentration, mTORC1 inhibition improves tubular autophagy, mitophagy, and fibrotic change in both rats and patients with diabetic nephropathy [93, 141]. Additionally, it was shown that inhibition of autophagy can markedly reverse the protective effect of mTORC1 downregulation on tissue damage and fibrotic change in a rat model of diabetic nephropathy [142]. mTORC1 downregulation lowers TGF-*β* expression and attenuates TGF-*β*-mediated collagen deposition and epithelial-mesenchymal transition in diabetic nephropathy (Figure 2) [136]. Even mTORC1 inhibition in the proximal convoluted tubule has been strongly implicated in the renoprotective effects of sodium-glucose cotransporter 2 (SGLT-2) inhibitors in diabetic nephropathy [136]. Recently, a meta-analysis of 38,723 individuals uncovered that using SGLT-2 inhibitors is significantly associated with reduced risk of acute kidney injury, end-stage renal disease (ESRD), and eGFR decline [143]. mTORC1 inhibition by sodium-glucose linked transporter 2 (SGLT-2) inhibitors was shown to protect against renal fibrosis and albuminuria and improve renal function in mice [136].

Furthermore, constitutive activation of mTORC1 abolished the renoprotective effects of SGLT-2 inhibition in diabetic nephropathy [136]. Likewise, mTORC1 inhibition was shown to be involved in the beneficial effects of losartan in diabetic nephropathy [144]. Losartan inhibited mTORC1 function in both glomeruli and podocytes of diabetic rats [144]. An increasing number of studies are indicating that mTORC1 possesses a critical role in the development and progression of diabetic nephropathy through perturbation of oxidative stress, autophagy, mitophagy, and fibrogenesis. Therefore, mTORC1 inhibition is of interest to attenuate renal failure in diabetic nephropathy.

## 5. Sestrin2 and RAAS System

Overactivation of the renin-angiotensin-aldosterone system (RAAS) has been implicated in the development and progression of diabetic nephropathy, and ARB/ACE inhibitor blockade is currently the mainstay of treatment [145]. Angiotensin II enhances oxidative stress and ER stress and increases albumin permeability in podocytes [146, 147]. Conversely, RAAS blockade improves proteinuria and podocyte viability and preserves renal function in diabetic nephropathy [148]. Similarly, RAAS inhibition can ameliorate renal tubular epithelial cell injury and prevent epithelial-mesenchymal transition in diabetic nephropathy [149].

It was shown that upregulation of Sestrin2 can effectively attenuate the detrimental effect of RAAS overactivity on cell viability; however, Sestrin2 does not modulate the release of renin, angiotensin, and aldosterone [150]. In addition, Sestrin2 knockdown deteriorates the effects of RAAS overactivity on cell viability [151]. Sestrin2 upregulation can modulate the toxic effect of angiotensin II on endothelial cells [151]. In particular, Sestrin2-mediated release of Nrf2 attenuates oxidative stress and inhibits angiotensin II-induced apoptosis of endothelial cells [152]. The protective effect of Sestrin2 against RAAS overactivity can increase its therapeutic value in diabetic nephropathy.

## 6. The Potential of Noncoding RNAs in Regulating Sestrin2 Signaling Pathway

Recently, it was shown that noncoding RNAs are involved in regulating gene expression, and their imbalance is observed in the pathogenesis of diabetic nephropathy [153]. Noncoding RNAs exert transcriptional and translational modifications and modulate signaling pathways in diabetic nephropathy [153]. It has been observed that several noncoding RNAs regulate Sestrin2 expression [154, 155]. For instance, microRNA-122 delivery or inhibition of miR-148b-3p and miR-199a-5p increased Sestrin2 expression in animal studies and cell culture in previous studies [154, 155]. Also, it has been uncovered that noncoding RNAs can alter Sestrin2 expression in diabetic nephropathy [8]. For instance, miR-4756 downregulated Sestrin2 in diabetic nephropathy, thereby accelerating disease progression [8]. Therefore, enhancing Sestrin2 expression for diabetic nephropathy by manipulating the noncoding RNA network may be a feasible therapeutic method in the future.

## 7. Conclusion

Sestrin2 can modulate several molecular signaling pathways involved in the pathogenesis of diabetic nephropathy. For instance, Sestrin2 enhances Nrf2-mediated antioxidant defense and PGC-1*α*-mediated mitochondrial biogenesis. Furthermore, by activating AMPK and regulating GATOR2/GATOR1/RagB axis, Sestrin2 attenuates mTORC1-dependent inhibition of autophagy. ER stress can activate Sestrin2 through PERK/eIF2*α*/ATF4; in return, Sestrin2 alleviates uncontrolled ER stress. As the primary molecule of several signaling pathways, upregulation of Sestrin2 can provide a wide variety of protective effects in diabetic nephropathy compared with each one of the downstream molecules. Based on the results of previous studies, the function of Sestrin2 is impaired in diabetic nephropathy, and restoration of its function can reverse a series of molecular alterations, improving renal function, albuminuria, and structural damage.
